# Gait, posture and cognition in Parkinson's disease

**DOI:** 10.1590/s1980-5764-2016dn1004005

**Published:** 2016

**Authors:** Alessandra Ferreira Barbosa, Janini Chen, Fernanda Freitag, Debora Valente, Carolina de Oliveira Souza, Mariana Callil Voos, Hsin Fen Chien

**Affiliations:** 1 Physical Therapy, Occupational Therapy and Speech Therapy Department, University of São Paulo School of Medicine, São Paulo, SP, Brazil.; 2 Movement Disorders Clinic of Hospital das Clinicas of the Department of Neurology. University of São Paulo School of Medicine, São Paulo, SP, Brazil.; 3 ReMove. Rehabilitation of Movement Disorders Research Group.; 4 Institute of Orthopedics and Traumatology of Hospital das Clínicas of University of São Paulo School of Medicine, São Paulo, SP, Brazil.

**Keywords:** gait, cognition, Parkinson's disease, fall, gait assessment

## Abstract

Gait disorders and postural instability are the leading causes of falls and disability in Parkinson's disease (PD). Cognition plays an important role in postural control and may interfere with gait and posture assessment and treatment. It is important to recognize gait, posture and balance dysfunctions by choosing proper assessment tools for PD. Patients at higher risk of falling must be referred for rehabilitation as early as possible, because antiparkinsonian drugs and surgery do not improve gait and posture in PD.

## Abstract (pt)

Distúrbios da marcha e perda de controle postural são as causas principais de queda e incapacidade na doença de Parkinson (DP). A alteração cognitiva desempenha um papel importante para a perda do controle postural e aumenta o risco de quedas; ela também interfere no cuidado e tratamento dos distúrbios da marcha. É importante reconhecer os transtornos da marcha, postura e equilíbrio escolhendo instrumentos de avaliação adequados para identificar os pacientes que tem maior risco de quedas e referi-los para reabilitação o mais precocemente possível, porque os medicamentos e a cirurgia não melhoram a marcha, nem a postura de pacientes com DP.

## INTRODUCTION

Patients with Parkinson disease's (PD) experience 62% more falls than patients with other neurological diseases. Falls are correlated with multiple factors, including postural, gait and cognitive dysfunction. Postural control and gait dysfunction may occur in early stages of PD and are characterized by postural instability, reduced arm swing, shorter path length, and loss of disassociated arm and trunk movements during gait.

Postural control requires the integration of three systems: visual, somatosensory and vestibular; and adaptation to continuous environmental changes.^1^ However, this control may be disrupted by concurrent performance of a cognitive or another motor task.^2^ According to Hausdorff et al.,^3^ attention deficits lead to major changes in gait variability and stability. When PD patients accomplish a predetermined combined gait task, they are more likely to reduce walking speed and stride length and exhibit more freezing episodes, compared to performing single tasks. Single tasks are controlled by the basal ganglia, which are affected in PD. Dual tasks activate the frontal brain area and require voluntary/conscious control.^4^

Postural balance is not a static condition, but a dynamic activity that requires constant neuromuscular responses.^5^ This also holds true both in resting posture and while moving the body, e.g. walking, which involves shifting the center of gravity. Environmental demands must be identified by processing sensory inputs.^6^

Balance disturbances result from shifts in the center of gravity which may have an internal (e.g. natural oscillation of the body, limb movement) or external (a sudden push or braking car) origin. In this context, when there are deficits in the interpretation and integration of sensory systems, in planning the right responses or in the recruitment of the correct muscles to keep the center of gravity within the support base limits, the individual becomes susceptible to falls.^5,7^

The interaction between sensory, cognitive and motor systems is very important in PD patients. The disease can disrupt all these systems and, consequently, increase the risk of falls.^8,9^ Postural instability is a motor manifestation of multiple causes and decreases the quality of life of PD patients. Also, it is not easily treatable by antiparkinsonian drugs and/or surgery.^10,11^ Christofoletti et al.^9^ investigated which factors are important predictors of gait disturbance and reduced mobility and noted that the major predictor of disability was poor postural balance.

Cognitive impairment, often manifested initially as executive dysfunction, affects both integration of sensory information and the motor planning required for maintaining balance, especially in dynamic activities such as walking.^12^

Therefore, appropriate assessment of gait and balance impairments are imperative and should also include the evaluation of executive function. Detection of cognitive and gait disturbances in PD patients may improve the quality of life of this population and reduce their risk of falls by earlier intervention and adequate therapeutic strategies.

## POSTURE AND GAIT MOTOR ASSESSMENT IN PD

There are several rating scales, questionnaires, and timed tests that assess posture, gait and balance of PD patients, but few include more complex tasks that reflect environmental demands. We recommend careful choice of measurement instruments and the association of two or more tests for better evaluation of PD gait and balance.^7,8,13^ Table 1 summarizes the instruments outlined below.

**Table 1:** Questionnaires, Instruments and Clinical Tests for Balance and Posture Assessment in PD.

Questionnaire/Instrument/Clinical Test	Domain
1. Static and Dynamic Computerized Posturography	Static and Dynamic Balance/Posture
1a. Dynamic Computerized Posturography	Dynamic Balance/Posture
1b. Sensory Organization Test (SOT)	Static Balance/Posture
1c. Multidirectional body movements on force platform	Dynamic Balance
1d. Balance Master System	Dynamic Balance
2. Timed Up and Go test	Gait (rising, walking, turning)
3. Berg Balance Scale	Static and Dynamic Balance
4. MiniBESTest	Static and Dynamic Balance/Gait

The assessment of static and dynamic balance, with or without the association of a cognitive task, is of great importance. Computerized posturography is an exam that assesses, using a force platform, the reaction forces that the body exerts on the ground and the trajectory of the center of pressure (COP). The information provided indicates the patient's neuromuscular responses under the several conditions cited above. This technology can be used under different conditions, e.g. open eyes, visual and/or vestibular deprivation, narrow base of support and with a cognitive or motor task concomitantly.

Several studies have sought to elucidate the static balance of PD patients using posturography technology, but results remain controversial.^14-17^ Some authors reported that PD patients show higher COP oscillation compared with age-matched healthy volunteers. Other authors have shown that PD patients oscillated less or within the same range as the control group. It is difficult to compare these studies since they have employed different methodologies concerning PD staging, freezing frequency, falls history, measurement instruments, variables and tasks.^10,18,19^

Nevertheless, more studies seem to agree that, in the presence of sensory disturbances, patients with PD have higher COP sway, and, consequently, greater postural instability.^8,10,20-22^ A recent study by our group^10^ found that PD patients oscillated more than controls even under basal condition (open eyes). However, this oscillation was higher when the patient experienced vision deprivation. Static balance was also assessed under dual task conditions, in a standing position, and with a verbal fluency task. We noted that the association of the cognitive task resulted in higher disruption of balance compared to sensory disturbance without the association of a cognitive task. These results were consistent with the findings of Marchese et al.^23^ The authors associated the standing position with a cognitive task (subtraction), comparing to baseline and to a dual motor task (standing and performing a finger sequence simultaneously). Fernandes et al.^22^ published a similar study and replicated these results. They demonstrated that a cognitive task exposed the patient to greater instability, even in static postures.

While this effect has been seen under static posture conditions, higher cognitive demands, such as verbal fluency, arithmetic or an activity that requires more complex motor planning, can also influence performance on dynamic balance tasks.^7^ Dynamic posturography measures how patients respond to mechanical or sensory disturbances. It is possible to identify responses during a predetermined task, assessing motor skills and balance, or only observing responses to external disturbance. The assessment of postural instability represents a major challenge for science, because it includes several complex systems. When sensory integration is altered, and/or cognitive function is impaired, motor responses become ineffective, leading to postural control dysfunction.^24^

The Sensory Organization Test (SOT) is a postural control assessment method. It entails the manipulation of visual, vestibular and proprioceptive neuromuscular information and evaluates how these systems respond. It is composed of six conditions for the triad of balance: vestibular, multisensory or physiological abnormalities. This technique involves tilting the surface or the visual surroundings under six conditions. The first three conditions provide a basic measurement of stability, standing with eyes open or closed on a static force plate surface, with visual surroundings moving or fixed. In conditions four to six, the patient experiences the disruption of somatosensory information, with a moving platform surface.^25^

The use of SOT in association with the MiniBESTest can identify 47% more deficits in anticipatory postural control, postural responses and gait, and sensory integration than the use of SOT assessment alone. This finding suggests the importance of retaining posturographic methods to evaluate balance.^7^

Another possibility for assessing dynamic stability is by evaluating responses after platform surface movements. It is possible to analyze COP displacement (as in static posturography) and assess ankle and hip balance strategies, integrated with electromyography. In one study, dynamic testing showed a decrease in the number of falls with increased sensorimotor strategies in 12 patients after bilateral deep brain stimulation (DBS) of the subthalamic nucleus.^26^ It was also confirmed that trunk movements and muscle activity were equally present in PD patients and age-matched controls. However, in the PD group, leg muscle responses, and also medial deltoid and masseter activities were increased.^27^

Dynamic posturography measures balance while the patient is standing on a platform with a cylindrical base of support. The main objective of this method is to continuously compensate self-induced disturbances. One study showed that patients with poor performance in functional balance tests also had higher body sway and poorer reactive postural responses on posturography compared to patients that had normal adjustments on the pull test, which denoted a close relationship between clinical tests and posturography.^16^

Multidirectional body movements on the force platform is another useful technique for assessing the risk of falls in PD. In one study, patients stood on a fixed platform surface and moved their body in several directions, without stepping, following the directions established by the computer screen monitor, known as Limit of Stability (LOS). Patients with a history of falls had lower reaction time and velocity. The performance on LOS was correlated with the performance on functional tests, including the Unified Parkinson Disease Rating Scale, Timed Up and Go test, and Berg Balance Scale (BBS).^17^

The Balance Master System is a computerized posturography instrument designed for balance assessment. It evaluates body oscillations under different conditions (in orthostatic position to assess the LOS, SOT, to perform tasks related to daily activities and movement skills: sit-to-stand, tandem walk, turn, step over a box). It is also possible to develop a training sequence for therapeutic purposes to challenge patient's limitations. Abilities requiring strength, balance, mobility and cognitive processing to perform certain tasks, such as stepping over, are a challenge for PD patients.^28,29^

The Timed Up and Go test is an appropriate tool for assessment of functional mobility and focuses more on executive function, compared to the BBS and Dynamic Gait Index (DGI) questionnaires.^31^ TUG measures the time required to perform a sequence of activities, including the sit-to-walk, transfer, straight walking, turning and the walk-to-sit. The Dual task TUG has been shown as a good assessment for measuring gait and executive function. According to Christofoletti et al.,^32^ the performance of PD patients is influenced by dual tasks, when the ability to adapt to environmental changes is affected.

The Berg Balance Scale is widely used to assess dynamic and static balance in elderly populations.^33^ BBS is extensively used to assess balance in PD patients, with good validity and reproducibility. However, in some cases, high scores on BBS do not assure normal gait parameters in dual tasks, nor the absence of freezing.

The MiniBESTest^34^ assesses balance using 14 tasks including the TUG, Push and Reach test, gait speed, negotiating obstacles, and turning. According to Mak et al.,^35^ scores lower than 19 are good predictors of recurrent and future falls in PD patients.

The choice of proper clinical balance assessment instruments and their association with static and/or dynamic postural tests contributes to better identification of patients with higher risk of falls. But which tests should be chosen? Bloem et al.^36^ reviewed many clinical tests for the Movement Disorders Society Rating Scales Committee. Although there are several instruments that adequately assess freezing of gait and balance confidence in PD, most clinical rating scales for gait, balance, and posture perform suboptimally. The authors recommend future development of a PD-specific, easily-administered, comprehensive gait and balance scale that separately assesses all relevant constructs with good clinimetric properties.

## THE INFLUENCE OF EDUCATION IN PD

The concept of an education-augmented cognitive reserve has been extensively investigated in studies focusing on dementia, normal aging, and PD.^37^ However, few studies have explored the relationship between education and motor outcomes. The possible mechanisms that might explain this relationship include: (1) greater education-associated cerebral volumes that are more resilient to neurodegenerative changes; (2) more efficient recruitment of alternative brain networks that may be used for neurological function; or (3) enhanced brain repair/recovery mechanisms.^37^

Kotagal et al.^38^ conducted a cross-sectional clinical imaging study of 142 subjects with PD. All subjects underwent [^11^C] dihydrotetrabenazine PET to confirm nigrostriatal dopaminergic denervation and also brain MRI to estimate adjusted cortical gray matter volume (GMV). After adjusting for possible confounders, including cognitive and dopaminergic covariates, as well as nonspecific neurodegeneration covariates (age, disease duration, and total adjusted cortical GMV), lower years of education remained a significant predictor of higher total MDS-UPDRS motor scores. Educational level was inversely associated with white matter (WM) hyperintensities. Higher educational attainment is associated with lower severity of motor impairment in PD, and this association may reflect an extranigral protective effect upon WM integrity.

These results are also consistent with previous studies that have shown inverse correlations between balance performance in PD and educational attainment.^39,40^

Souza et al.^40^ investigated the influence of educational status (number of years of formal education) on executive function tasks and balance. PD patients and healthy elderly controls were asked to perform the Trail Making Test (TMT) and BBS. Participants with lower educational status (both PD and control groups) performed worse on the TMT Part B than those with higher educational status. Within the PD group, the less-educated patients scored worse on the BBS than the more-educated group. This finding may be attributed to higher cognitive reserve in those patients with PD who had more years of formal education.

Cognitive enrichment fosters the development of neuroplasticity, which permits the maintenance of cognitive function even in a person with brain pathology. Cognitive resources, such as visual perception, memory, divided attention, coordination, motor sequencing, and executive function, help to reduce the risk of falls and to compensate for balance impairment in older adults.^41,42^

Voos et al.^41^ demonstrated that, in healthy elderly people, both poor executive function performance (assessed by the TMT) and Low Educational Status (assessed by self-reported years of formal education) may be related to lower functional balance scores on BBS. According to the literature, the influence of formal education on executive function is well established.^39-43^ If educational status influences the development of executive function during lifespan, and executive function is related to balance and gait,^43-45^ it follows that educational status may also have some influence on balance and gait.

## CHOLINESTERASE INHIBITOR AND PD GAIT

The pedunculopontine nucleus (PPN) is located in the caudal mesopontine tegmentum and provides the majority of cholinergic inputs to the thalamus, with projections to the striatum, cerebellum, and brainstem. It also has connections with the basal ganglia nuclei, specifically the substantia nigra, subthalamic nucleus, and globus pallidus interna. The PPN and nucleus basalis of Meynert degenerate in PD and the cholinergic loss is associated with cognitive impairment. PD dementia and dementia with Lewy Bodies present fluctuation in conciousness, which can be improved by cholinesterase inhibitors (CI). This fact suggests that cholinergic loss participates in the cognitive spectrum associated with Lewy body deposition and α-synuclein accumulation.^46^

Falls are related to cognition and may be caused by similar neurochemical disturbances. Evidence from imaging studies supports the idea that degeneration of the cholinergic system in PD may be responsible for a number of motor and non-motor symptoms, including cognitive dysfunction, depression, falls and postural instability. Moreover, cholinergic degeneration may also be associated with gait dysfunction. Greater postural instability, which is related to falls, is correlated with worse scores on tests associated with attention and executive function. The interaction between attention and gait may be observed during dual tasking and is endorsed by reports of CI reducing the risk of falls in PD patients.^46^

In animal models, cholinergic–striatal disruption of attentional–motor pathways in the basal forebrain is a major cause of falls. The association between cortical and subcortico-mesencephalic cholinergic deficits in patients with PD without dementia with high-level gait disorders (such as freezing of gait), postural instability, falls, coupled with dysexecutive syndrome and apathy, require further research.^47^

Gait speed in PD is also correlated with cholinergic function. Bohnen et al.,^48^ observed that a PD subgroup with preserved cortical cholinergic innervations displayed no significant slowing of gait speed compared with non-PD control subjects. The multisystem hypocholinergic effect on gait speed was driven by basal forebrain but not by PPN-thalamic denervation effects, and the latter is associated with postural control. Alterations in cognitive function are linked to gait disturbances, while gait speed reduction predicted cognitive decline in initially unimpaired older adults. According to the authors, the mechanism of slowing of gait in PD can be conceptualized as a clinical state, in which preattentive striatal degradation is initially supplemented by increasing cognitive control mediated by cortical cholinergic mechanisms. As compensatory cholinergic systems degenerate, gait speed is reduced.

Recent trials with high-dose rivastigmine in non-demented PD patients indicated that the drug can improve gait stability and might reduce the frequency of falls. However, the drug had no significant effects on episodes of freezing of gait or on the neuropsychological outcomes assessed.^49^ This finding contradicts a previous meta-analysis conducted by Pagano et al.,^50^ in which CI proved an effective treatment for cognitive impairment in patients with PD but failed to reduce the risk of falls. This class of medications impacted positively on global assessment and behavioral disturbance without significantly affecting motor function scales. In fact, an earlier double-blind, placebo-controlled study of PD patients with pre-dementia apathy showed that rivastigmine treatment was associated with a lower overall apathy score and caregiver burden, as well as greater intellectual curiosity and action initiation.^51^

Future trials might answer the question of how falls relate to cholinergic and attention function. Many researchers have investigated the effect of PPN stimulation in PD patients because of previous reports of motor benefits. Mestre et al.^52^ reported their result of long-term unilateral PPN DBS and showed an initial benefit of PPN stimulation for PD gait-related symptoms but after 4 years this improvement was not sustained. The authors highlighted some limitations to their findings including technical issues and outcome assessment. However, to what extent does degeneration of PPN cholinergic neurons affect gait disturbances in PD ? Moreover, the correlations between the various patterns of cholinergic denervation and clinical phenotypes in PD have not been studied.

## IMPLICATIONS FOR REHABILITATION IN PD

Nocera et al.^30^ conducted a study in which PD patients performed a 10-week home exercise program targeting abdominal muscles. The program included squats, calf raises, and step-up exercises. The authors showed significant improvement in four to six SOT conditions, with unstable surface in eyes open and closed conditions. Patients improved balance with home exercise, contributing to maintaining postural stability and reducing the risk of falls. Therefore, focusing on motor training can also improve dual-task performance and balance.

The dual task can influence and, consequently, the risk of falls in PD patients. Firstly, there was the assumption that when the patient performed two simultaneous cognitive and motor tasks, performance would be impaired and there would be an increase in the number of falls. Therefore, such situations were avoided in therapy sessions. However, recent studies have shown that the subcomponents of these activities can serve as cues, helping patients in their locomotion. Mirelman et al.^53^ trained PD patients on a treadmill associated with virtual reality. They found a positive impact when patients performed both training tasks simultaneously, with decreased risk of falls and greater gait stability. This result favors dual task training in PD patients.

Plotnik et al.^54^ demonstrated that cognitive-motor complexity act together to control gait in PD patients suffering from fluctuations in motor responses. There was a pronounced effect of dual task conditions on gait variability, asymmetry and bilateral coordination. Other studies report that there is improvement of mobility and cognitive function, as well as a reduction in the risk of falls, when motor and cognitive activities are integrated. These affirmations emphasize the importance of dual-task training and the development of gait rehabilitation programs. Moreover, dual tasks are part of our activities of daily living.^55,56^

Strategies to improve gait depend on the ability to use attention and mental images, which rely on cognitive ability. These strategies include visual and auditory cues.^57^ Recent studies show the benefits of these cues for decreasing the risk of falls and reinforce the hypothesis that patients with PD require more attention for motor task performance.

In conclusion, rehabilitation contributes to the improvement of gait and falls prevention. When using the cues, there is explicit, semantic and episodic memory (declarative) demand to compensate for the loss of the implicit memory, which is affected in PD. Many studies have also shown benefits of combined cueing (auditory and visual cues). Positive motor impact has been observed in gait parameters (length, time and cadence).^58^
